# Cost-effectiveness of a multicomponent primary care program targeting frail elderly people

**DOI:** 10.1186/s12875-018-0735-4

**Published:** 2018-05-16

**Authors:** Franca G. H. Ruikes, Eddy M. Adang, Willem J. J. Assendelft, Henk J. Schers, Raymond T. C. M. Koopmans, Sytse U. Zuidema

**Affiliations:** 1 Radboud Institute for Health Sciences, Department of Primary and Community Care, Radboud university medical centre, Nijmegen, the Netherlands; 2 Radboud Institute for Health Sciences, Department for Health Evidence, Radboud university medical centre, Nijmegen, the Netherlands; 3 Joachim and Anna, Centre for specialized geriatric care, Nijmegen, the Netherlands; 4 Department of General Practice and Elderly Care Medicine, University of Groningen, University Medical Centre Groningen, Groningen, Netherlands

**Keywords:** Cost-benefit analysis, Frail elderly, Delivery of health care, Integrated, Activities of daily living, Primary health care

## Abstract

**Background:**

Over the last 20 years, integrated care programs for frail elderly people aimed to prevent functional dependence and reduce hospitalization and institutionalization. However, results have been inconsistent and merely modest. To date, evidence on the cost-effectiveness of these programs is scarce. We evaluated the cost-effectiveness of the CareWell program, a multicomponent integrated care program for frail elderly people.

**Methods:**

Economic evaluation from a healthcare perspective embedded in a cluster controlled trial of 12 months in 12 general practices in (the region of) Nijmegen. Two hundred and four frail elderly from 6 general practices in the intervention group received care according to the CareWell program, consisting of multidisciplinary team meetings, proactive care planning, case management, and medication reviews; 165 frail elderly from 6 general practices in the control group received usual care. In cost-effectiveness analyses, we related costs to daily functioning (Katz-15 change score i.e. follow up score minus baseline score) and quality adjusted life years (EQ-5D-3 L).

**Results:**

Adjusted mean costs directly related to the intervention were €456 per person. Adjusted mean total costs, i.e. intervention costs plus healthcare utilization costs, were €1583 (95% CI -4647 to 1481) higher in the intervention group than in the control group. Incremental Net Monetary Benefits did not show significant differences between groups, but on average tended to favour usual care.

**Conclusions:**

The CareWell primary program was not cost-effective after 12 months. From a cost-effectiveness perspective, widespread implementation of the program in its current form cannot be recommended.

**Trial registration:**

The study was registered in the ClinicalTrials.govProtocol Registration System: (NCT01499797; December 26, 2011). Retrospectively registered.

## Background

Frail elderly account for a disproportionally large share of healthcare costs, spending over $70,000/year in 2011 in the United States, with particularly high expenditure on in-patient and post-acute care [1, 2]. In the Western world, the prevalence of frailty - a state of increased vulnerability to adverse outcomes through a complex interplay of physical, psychological, social and environmental factors [3] – will even increase due to population ageing, since frailty is thought to be present in 10% of people aged ≥65 years up to 25%–50% of people aged ≥85 years [4, 5]. Western countries are forced to adapt their healthcare policies addressing frail elderly in order to achieve cost reductions in health and social services and maintain financial sustainability.

Proactive integrated care programs, addressing the complex and interacting healthcare and welfare needs, are thought to have the potential to prevent adverse outcomes and lower healthcare costs [6, 7]. However, results so far have shown merely modest, inconsistent results regarding their effectiveness and efficiency [8–12]. Some studies pointed out the potential to prevent hospitalization and nursing home admissions [10, 11, 13], but accompanying increases in home care and social services use might impede overall cost savings [8, 10, 14]. Formal economic evaluations of integrated programs targeting frail elderly are scarce [15, 16]. Moreover, heterogeneity between studies regarding target population (age, low or high risk of functional decline), context (home-, primary care- or institution based), and intervention components hinder comparability and generalizability. Moreover, results of economic evaluations need to be interpreted in the light of national contexts [17].

In the Netherlands, the Dutch Ministry of Health, Welfare and Sports initiated the National Care for the Elderly Program in 2008, in which over 650 organizations in health, welfare and housing work together in eight regional networks led by academic medical centres to improve care for elderly people with complex care needs. As part of this program, we developed the CareWell primary care program that aimed to reduce functional decline, institutionalization, and hospitalization of community-dwelling frail elderly. Although effectiveness of the program could not be demonstrated [18], the program might theoretically save overall costs and, depending on the trade-off between costs and effects, might be cost-effective. Therefore, we conducted a separate economic evaluation to answer the following research questions:

- What are the differences in health care costs between participants receiving care according to the CareWell primary care program and those receiving care as usual?
- Is the CareWell primary care program cost-effective from a healthcare perspective after 12 months?

## Methods

### Design

This economic evaluation from a healthcare perspective was performed alongside a cluster controlled effectiveness study with a follow-up of 12 months. Design, methods and outcomes of the effectiveness study have been reported elsewhere [18].

### Setting and participants

The study was conducted between September 2011 and September 2012 in 12 general practitioner (GP) practices in the region of Nijmegen, the Netherlands. After informed consent, frail elderly aged ≥70 years were included with the use of the EASY-Care TOS instrument [19]: First, GPs use prior knowledge to subdivide ‘not frail’ from ‘(possibly) frail’ elders. Then, trained nurses perform a comprehensive geriatric assessment of (possible) frail elders during a home visit. Last, GPs and nurses weigh all signs into a final frailty judgment. Exclusion criteria were institutionalization, and/or critical or terminal illnesses. Details on the recruitment and informed consent procedures have been reported previously [18, 20].

### Intervention

In brief, the CareWell primary care program consisted of four key components: 1) multidisciplinary team (MDT) meetings, 2) proactive care planning, 3) case management, and 4) medication reviews.

Each practice assembled a MDT consisting of a general practitioner (GP), practice nurse(s) and/or community nurse(s), an elderly care physician (ECP) [21], and a social worker with elderly care expertise. Each participant was discussed in a MDT meeting at least half-yearly, more often if needed. Meetings were planned every 4–8 weeks. Tailor-made proactive care plans, based on the individual health-related problems and goals as assessed with the EASY-Care TOS [19], were formulated for each participant on enrolment in the program and revised after discussion in a MDT meeting at least every 6 months. A case manager, either a nurse or social worker, was assigned to each participant. They were responsible for care planning and coordination, patient-support in goal setting and self-management, and caregivers support. Last, the GP and nurse conducted a yearly medication review in collaboration with a pharmacist for each participant with polypharmacy (use of ≥5 chronically prescribed drugs).

Professionals received financial reimbursement to cover time-investment and overhead costs.

### Usual care

In the Netherlands, GPs provide continuous, person-centred care to community-dwelling frail elderly, facilitated by the use of high-standard electronic medical records and patient panels, defining the population under care [22, 23]. GPs often collaborate with practice and/or community nurses. Moreover, elderly care physicians, i.e. medical practitioners that are specialized as primary care experts in geriatric medicine, increasingly operate (as consultants) in the care for community-dwelling frail elderly [21]. However, the coordination between GPs, other primary and specialist care providers, and home care and community services is often perceived to be insufficient, leading to a fragmented delivery of care [24].

GPs in the usual care group were explicitly asked to decline new relevant inter professional collaborations during the intervention period. No restrictions on pre-existing collaborations between GPs and (practice) nurses were imposed.

### Outcome measures

Dependence in functioning in (instrumental) activities of daily living (measured with the Katz-15 [25] change score, i.e. follow-up score minus baseline score) and health-related quality of life (measured with the EuroQol five-dimensional three-level instrument (EQ-5D-3 L) [26]) were collected at baseline and at follow-up after 12 months by structured interviews by trained nurses. The Katz-15 score ranges from 0 to 15 points with higher scores indicating more dependence in (instrumental) activities of daily living. The EQ-5D-3 L instrument is a ‘preference-based’ measure of health status [27], that defines health-related quality of life according to five dimensions (mobility, self-care, usual activities, pain/discomfort, and anxiety/depression) at three levels (no problems, some problems, severe problems) [26]. In line with the guidelines of the National Care for the Elderly Program, we used the modified EQ-5D + C-3 L instrument that includes cognitive functioning as an additional dimension, with a similar operationalization at three levels [28]. To date, to the best of our knowledge, there is no validated weighting formula for the EQ-5D + C-3 L. Utilities, reflecting the relative desirability of each health state, were thus calculated for the EQ-5D-3 L, without the cognitive dimension, using the Dutch tariff [29]. EQ-5D-3 L scores range from − 0.33 to 1.00, with a higher score indicating a higher health status. Quality Adjusted Life Years (QALYs) were then calculated by multiplying the utilities by the amount of time spent in a particular health state. 1 QALY represents 1 year in perfect health [29].

### Healthcare utilization costs and intervention costs

We assessed intervention costs and healthcare utilization during the follow up period. [17] An overview of the healthcare cost variables, prices per unit and sources are presented in Table 1.

**Table 1:** Overview of the cost variables, sources, and cost prices per unit

Cost variable	Source of variable	Cost price per unit (in Euros)
Healthcare utilization costs:		
GP care^a^ (per contact):		
Consultation	Structured interview	28
Consult > 20 min	Structured interview	56
Home visit	Structured interview	43
Home visit > 20 min	Structured interview	72
Consultation by phone	Structured interview	14
Prescription refill	Structured interview	14
GP care, out of office hours^b^ (per contact)	Structured interview	101
Home care (per hour)	Structured interview	35
Domestic care (per hour)	Municipality registries	12,5
Hospital care, inpatient (per day)	Structured interview	457
Hospital care, outpatient (per contact)	Structured interview	72
Nursing home (per day)	Structured interview	238
Care home (per day)	Structured interview	90
Day care (per day)	Welfare organization registries	45
Physiotherapy (per contact)	Structured interview	36
Medication^c^	Electronic patient file	n/a
Intervention costs (per hour):	Time registrations	
General Practitioner^b^		103
Practice nurse^d^		30
Community nurse^d^		27
Social worker^d^		32
Elderly care physician^b^		103
Pharmacist^b^		85

Sources of cost prices per unit:

^a^Dutch guideline for costing research[30]

^b^Dutch Healthcare Authority

^c^Royal Dutch Society for Pharmacy[31]

^d^Collective Agreements

Intervention costs regarding time spent on team meetings, care planning, case management, and medication reviews were assessed by instructing practice and/or community nurses and social workers to fill in monthly time registration forms at participant level. To stimulate uniformity in and compliance with time registrations, structured timesheets with written instructions were sent each month as reminders. GPs and ECPs estimated their mean time spent on the intervention per GP practice, from which invested time per participant was calculated. Pharmacists estimated a time investment of 30 min per participant per medication review.

Healthcare utilization variables, i.e. GP care, hospital care, institutionalization (i.e. nursing home admission, care home admission), home care, and physiotherapy were individually assessed at baseline and follow up through a structured interview by the nurse. Data on domestic care and day care were individually extracted from registries from the municipality of Nijmegen and welfare organizations. Last, data on medication costs (both reimbursed and non-reimbursed) were individually extracted from the electronic patient files (EPF).

Costs were calculated by multiplying volumes of care with their corresponding unit prices. In calculating costs of time invested by practice and/or community nurses and social workers we used their Collective Agreements. The thus generated hourly wages were raised with an estimated 45% for employers and overhead expenses and thus set on €30, €27, and €32 respectively [30]. We used hourly wages of €103, €103, and €85 in calculating costs of time invested by the GPs, ECPs, and pharmacists respectively, according to the fixed rates of the Dutch Healthcare Authority. Costs of healthcare utilization were valued according to the Dutch manual for costing research [30]. When no standardized unit cost prices were available, costs were derived from the Dutch Healthcare Authority. Medication costs were valued using prices of the Royal Dutch Society for Pharmacy [31], using minimum cost prices. All costs were presented in Euros, and indexed to the year 2011 using the consumer price index.

### Statistical analysis

Katz-15 change scores and EQ-5D-3 L scores were analyzed using mixed model multilevel analyses, accounting for clustering of participants within GP practices and correcting for those variables that differed between groups at baseline and correlated to the primary outcome, as well as for baseline Katz-15 and EQ-5D-3 L scores to account for regression to the mean [18]. Quality adjusted life years (QALYs) were derived from the EQ-5D-3 L using the trapezium rule (i.e. an approximation of the area under the QALY curve). Mean healthcare utilization costs were analyzed with descriptive statistics and compared between groups using multilevel mixed model analyses, adjusting for clustering of participants within GP practices and for relevant covariates. The incremental Net Monetary Benefit (iNMB) statistic was used to evaluate cost-effectiveness [32] and consequently used as the dependent variable in the mixed model. The iNMB prevents several statistical drawbacks of an incremental cost-effectiveness ratio and enables the use of multilevel regression techniques including covariates in a convenient way [17]. It indicates the monetary gains or costs of an intervention at explicit Willingness to Pay (WTP) thresholds per gained unit of effect. In formula: iNMB = (WTP * ∆ effects) – ∆ costs. An iNMB (and 95% lower-level confidence interval) greater than zero indicates significant cost-effectiveness of the intervention. We used five WTP thresholds per point improvement on the Katz-15 change score, i.e. €0, €5000, €10,000, €15,000, and €20,000, where no reference values were readily available. Six commonly used WTP thresholds per QALY were used: €0, €20,000, €40,000, €60,000, €80,000, and €100,000 [33].

## Results

### Participants

**Fig. 1:**
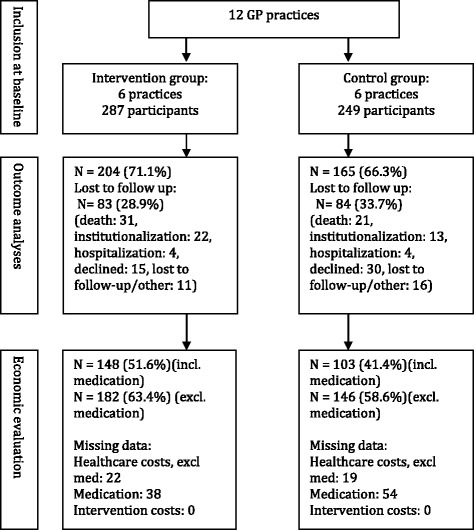
Flow diagram of participants

In total, 536 participants (287 in the intervention group resp. 249 in the control group) were included in the effectiveness study [18]. At baseline, participants in the intervention group significantly more often lived alone, had more health-related limitations in social functioning, more cognitive deficits, and more social disadvantage, but showed less complex care [18]. No significant between-group differences in baseline Katz-15 scores and EQ-5D-3 L scores were found. We had a loss to follow up of 28.9% participants in the intervention group and 33.7% in the control group, mainly due to death, institutionalization and declined consent for follow-up (Fig. 1). Additionally, we encountered a considerable number of missing cost variables, mainly medication cost data due to declined consent for use of EPF medication data and limited coverage of medication data in the EPFs. We adhered a complete case analysis with regard to missing values [34]. We analyzed costs and iNMB both with and without medication cost data, including 148 (51.6%) resp. 182 (63.4%) participants in the intervention group and 103 (41.3%) resp. 146 (58.6%) participants in the control group (Fig. 1), and considered the analyses including medication costs as the primary analysis. Participants included in the economic evaluation had a lower frailty index. This frailty index was calculated based on the accumulation of deficits in health (symptoms, morbidities, and/or functional abilities), and was used as an extra indicator of frailty next to the EasyCare-TOS [28]. It theoretically ranges from 0 (no indication of frailty) to 1 (extreme frailty), though frailty index scores in similar studies typically culminate at 0.7.Therefore, in addition to the covariates included in the effectiveness analysis, the frailty index was included as a covariate in this economic evaluation.

### Outcome measures

At 12 months, we found no significant differences in functional dependence (adjusted mean difference of 0.37, 95% CI -0.1 to 0.8) nor QALYs (adjusted mean difference of − 0.031, 95% CI -0.1 to 0.0) between the intervention and control group, but the control group did show less functional decline (Table 2) [18].

**Table 2:** Costs of care in intervention and control groups 0–12 months (in Euros)

	Intervention group		Control group		Adjusted mean difference^b^ (95% CI)	*P* value
	Unadjusted mean^a^	SE^d^	Unadjusted mean^a^	SE^d^		
Outcome:						
Katz-15 change score^1^	0.80	0.13	0.50	0.16	0.37 (−0.10 to 0.80)	.10
QALY^2^	0.60	0.02	0.60	0.02	−0.03 (− 0.10 to 0.00)	.37
Intervention costs:	456	14	0	0	− 455 (− 512 to − 398)	<.001
Healthcare utilization costs, total:	10,125	983	8114	845	−1143 (− 4198 to 1912)	.46
GP care	163	13	169	18		
GP care, out of office hours	40	11	36	7		
Hospital care, inpatient	1557	510	1225	248		
Hospital care, outpatient	239	24	304	40		
Nursing home	943	399	198	118		
Care home	416	218	161	76		
Day care	422	102	342	101		
Home care	3712	423	2787	412		
Domestic care	1472	91	1417	113		
Physiotherapy	988	309	485	87		
Medication	1617	296	978	126		
Total costs^c^	10,576	983	8114	845	−1583 (− 4647 to 1481)	.31

^1^Katz-15 index (range 0 to 15); higher score indicates more functional dependence in (instrumental) activities of daily living

^2^Quality Adjusted Life Years (QALY), as derived from the EQ-5D-3 L.

^a^Unadjusted means, analyzed with descriptive techniques

^b^Multilevel mixed model analyses, accounting for clustering and covariates

^c^Total costs = intervention costs plus healthcare utilization costs

^d^*SE* standard error

### Healthcare utilization costs and intervention costs

Mean intervention costs, adjusted for clustering and relevant covariates, were €456 (95% CI -512 to − 398). In the intervention group, mean total costs, i.e. intervention costs plus healthcare utilization costs, adjusted for clustering and relevant covariates, were €1583 (95% CI -4647 to 1481) higher than in the control group. Mean adjusted healthcare utilization costs, i.e. without the intervention costs, were €1143 (95% CI -4198 to 1912) higher in the intervention group. Of the healthcare utilization variables, only medication costs differed significantly, although mean costs of hospitalization, institutionalization, home care and physiotherapy in the intervention group exceeded those in the control group (Table 2).

### Economic analysis

**Fig. 2:**
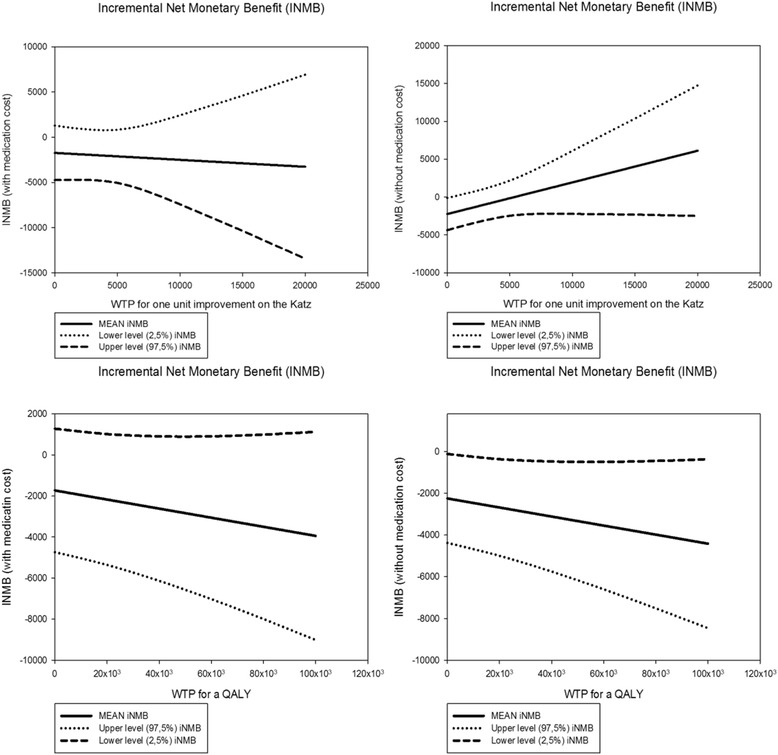
Incremental net monetary benefits (in Euros) against WTP for Katz-15 change score* and QALY. Upper panels show the incremental net monetary benefits (in formula: iNMB = (WTP * ∆ effects) – ∆ costs) against WTP for Katz-15 change scores; lower panels show iNMBs against WTP for QALY. All iNMBs are negative, i.e. the intervention does not provide value for money compared to usual care (not significant). Sensitivity analyses, excluding medication costs, underline the results (right panels)WTP = Willingness to Pay. QALY = Quality Adjusted Life Year, derived from the EQ-5D-3 L, based on the Dutch tariff [29] using the trapezium rule.* Improvement on the Katz-15 change score is indicated by a lower score, meaning less functional decline regarding (instrumental) activities of daily living

Figure 2 shows the iNMBs. It can be noticed that generally these iNMBs are negative, meaning that the intervention does not provide value for money compared to usual care, although the results are not significant. Sensitivity analysis, excluding medication costs, underlined these results.

## Discussion

In this cluster controlled study with a follow up of 12 months, healthcare utilization costs and cost-effectiveness of the CareWell primary care program was compared to usual care. Earlier, effectiveness of the CareWell program on daily functioning and quality of life could not be demonstrated [18]. In this study, we found no statistically significant differences between groups in total costs and healthcare utilization costs, with the exception of higher medication costs in the intervention group. Moreover, cost-effectiveness analyses showed no significant differences between groups, but tended to favour usual care.

There are some possible explanations for the absence of cost-effectiveness. First, there is still a lively debate on the concept of frailty and the right timing of interventions [35, 36]. Possibly, the targeted population was too heterogeneous or, in part, too frail to respond to the intervention. Second, the Katz-15 index, measuring daily functioning, might be too restricted to capture the effects of our heterogeneous intervention. Possibly, more person- or goal centred outcomes, e.g. goal-attainment scaling, might suit better [37]. Moreover, the sensitivity to change of the EQ-5D-3 L in frail elders might have been (too) low [38]. The concept of ‘capability wellbeing’ has recently been suggested as an alternative, more sensitive measure [33]. However, further work on the validity and value of these capability indices in economic evaluations is needed [33, 39]. Third, it is likely that more profound effects of the intervention only become apparent after a longer follow up period that exceeds the time needed for implementation, individual and organizational learning effects, and efficient multidisciplinary collaboration [40, 41]. This lag-time in effectiveness is presumed to be even more important in complex interventions like our program [40]. Awareness to these short-run inefficiencies that might have resulted from the time limits set by the National Care for the Elderly Program is needed. Last, the selection of motivated professionals in the intervention group might have limited the room for improvement in the delivered care and possibly led to higher costs due to more proactive care, irrespective of the CareWell program. The overall increased awareness to the health care needs of frail elderly in Western countries in the last two decades together with the Dutch high-quality primary care might have further reduced the contrasts between the CareWell program and usual care. Possibly, our program would show clearer effectiveness in less well managed healthcare settings [12].

Our results are in line with comparable integrated care programs aimed at frail elderly, performed in other contexts [14, 42]. More recently, three cost-effectiveness studies of integrated care programs from the National Care for the Elderly Program demonstrated no effects on functioning nor quality of life, at unchanged or higher total costs mainly due to increased GP care and intervention costs without (expected) decreases in hospital and long-term care costs, after 12–24 months [43–45]. However, Van Leeuwen et al. did find increasing effects at lower costs compared to usual care in the last 18–24 months of follow up [45]. Previously, Counsell et al. demonstrated similar decreased costs in their third year of follow up, mainly through a shift away from emergency and hospital services towards more-desirable chronic and preventive care expenditures [14]. This supports our assumption of a lag-time in effectiveness. The results of the cost-utility analyses of the recent other Dutch studies, finding low probabilities of the intervention increasing QALYs at lower costs, correspond with our results [45, 46]. However, only Van Leeuwen et al. performed a formal cost-effectiveness analysis [45]. Like us, they found low probabilities of the intervention being cost-effective.

This study has several strengths. First, we used a comprehensive approach to costing, including a wide variety of cost variables that were assessed at participant level, thus enhancing internal validity. Next, robust multilevel techniques were used in analyzing both differences in costs and net monetary benefits. Last, since we used only a limited number of exclusion criteria and included participants from heterogeneous GP practices, our results should be generalizable to the population of frail elders in the Netherlands and comparable high-quality primary care settings.

We also should consider some limitations. First, we were unable to include informal care costs, since informal caregivers’ willingness to participate was low and differed between groups. We were therefore not able to adhere to the societal perspective, as announced in our study protocol [20], but had to switch to a healthcare perspective. Since prior studies show contrasting results on the impact of informal care on total costs, the impact of this switch on our results is unclear [42, 45]. Next, since the extraction of data on healthcare use from external sources like healthcare insurance companies, as originally planned in the study design, was not possible, we had to collect these data through participants’ retrospective self-report. This could have led to recall bias. Different studies showed self-report after 12 months to be an appropriate, reasonably accurate method for obtaining a wide range of healthcare utilization data in elderly people [47, 48]. More salient events in general suffer less from memory decay and thus recall bias [49]. Seidl et al. for example found the recall bias of hospital admissions of elderly people not to be influenced by applying various recall periods, although the probability of correctly self-reporting a single event was higher using a shorter recall period [48]. However, less salient events such as GP contacts could lead to both under- and over-reporting, and show less accuracy in self-report [49, 50]. Also, time registrations used to calculate intervention costs might be biased due to inaccuracies. However, we have no reason to assume unequal distributions of these potential biases between the groups. Last, we had to deal with a considerable number of missing medication cost data that had to be considered missing not at random. However, our additional sensitivity analysis without medication costs did not reveal other results.

## Conclusions

After 12 months follow-up, no net monetary benefit of the CareWell program over usual care could be demonstrated.

This study adds to the currently scarce body of evidence regarding cost-effectiveness of integrated care programs targeting frail elderly. Future economic evaluations should account for pitfalls in their design with respect to the target population, outcome measures used, and adequate follow-up period. From a cost-effectiveness perspective, the CareWell primary program in its current form is not suited for widespread implementation.

## Abbreviations

ECP: Elderly care physician
EPF: Electronic patient file
EQ-5D-3 L: EuroQol five-dimensional three-level instrument
GP: General practitioner
iNMB: Incremental net monetary benefit
MDT: Multidisciplinary team
QALY: Quality adjusted life years
WTP: Willingness to pay
